# EdgeScaping: Mapping the spatial distribution of pairwise gene expression intensities

**DOI:** 10.1371/journal.pone.0220279

**Published:** 2019-08-06

**Authors:** Benafsh Husain, F. Alex Feltus

**Affiliations:** 1 Biomedical Data Science and Informatics Program, Clemson University, Clemson, SC United States of America; 2 Genetics and Biochemistry Department, Clemson University, Clemson, SC United States of America; 3 Center for Human Genetics, Clemson University, Clemson, SC United States of America; Politechnika Krakowska im Tadeusza Kosciuszki, POLAND

## Abstract

Gene co-expression networks (GCNs) are constructed from Gene Expression Matrices (GEMs) in a bottom up approach where all gene pairs are tested for correlation within the context of the input sample set. This approach is computationally intensive for many current GEMs and may not be scalable to millions of samples. Further, traditional GCNs do not detect non-linear relationships missed by correlation tests and do not place genetic relationships in a gene expression intensity context. In this report, we propose EdgeScaping, which constructs and analyzes the pairwise gene intensity network in a holistic, top down approach where no edges are filtered. EdgeScaping uses a novel technique to convert traditional pairwise gene expression data to an image based format. This conversion not only performs feature compression, making our algorithm highly scalable, but it also allows for exploring non-linear relationships between genes by leveraging deep learning image analysis algorithms. Using the learned embedded feature space we implement a fast, efficient algorithm to cluster the entire space of gene expression relationships while retaining gene expression intensity. Since EdgeScaping does not eliminate conventionally noisy edges, it extends the identification of co-expression relationships beyond classically correlated edges to facilitate the discovery of novel or unusual expression patterns within the network. We applied EdgeScaping to a human tumor GEM to identify sets of genes that exhibit conventional and non-conventional interdependent non-linear behavior associated with brain specific tumor sub-types that would be eliminated in conventional bottom-up construction of GCNs. Edgescaping source code is available at https://github.com/bhusain/EdgeScaping under the MIT license.

## Introduction

A fundamental goal of biology is to discover genetic relationships that coordinate the biochemical mechanisms underlying phenotype expression. A standardized way to contextualize these relationships is via gene co-expression networks (GCNs; also known as relevance networks [1]) that are mathematical graphs used to model complex global gene co-expression dependencies extracted from gene expression matrices (GEMs). In a GCN, nodes represent genes and edges are formed when a significant correlation exists between two genes across a set of Ribonucleic acid (RNA) expression profiles. The first reported GCN was by developed by Eisen et al. [2], and since then GCNs have been used for a several different analyses that are species-specific, including cancer studies [3–6]. A plethora of software tools and technologies have since been developed for the construction of GCNs, each using a different approach for identifying co-expression patterns. WGCNA [7], CLR [8], MRNET [9], RMTGeneNet [10], KINC [11], petal [12] and FastGCN [13] are few of the more broadly utilized techniques.

A limitation to discovering genetic correlation is that a GCN edge can only be detected if it was spatiotemporally present in the input sample set and is detectable above noise. Fortunately, millions of gene expression profiles containing rare gene expression relationships from diverse experimental conditions are available in public databases such as NCBI Short Read Archive [14] and Gene Expression Omnibus [15] databases, EBI ArrayExpress database [16], The Cancer Genome Atlas [17], Genotype-Tissue Expression [18], LINCS [19], ENCODE [20], and many others. In order to minimize confounding biological noise as datasets become deeper and more diverse, we have previously demonstrated that sorting multi-modal pairwise gene comparisons prior to a correlation test reveals condition-specific gene co-expression patterns as implemented in KINC software [11].

While much progress has been made in GCN construction, the current GCN construction strategies (a) are computationally intensive and are not scalable to GEMS with millions of samples; (b) do not detect non-linear relationships missed by correlation tests; and (c) do not place genetic relationships in a gene expression intensity context. In this study, we took a step back and engineered an algorithm called EdgeScaping that addresses all of the above limitations.

EdgeScaping is a novel technique to convert gene-gene edge data into an image format that substantially reduces the dimensionality of the data. This feature reduction technique is highly scalable to increases in GEM size. Further, converting gene expression data into an image permits one to leverage advanced image analysis techniques inspired by deep learning to detect non-linear features and relationships between edges that were previously undetected. Coupling image based data with an efficient deep learning algorithm, EdgeScaping implements a novel non-linear feature selection model to quickly and efficiently classify all the potential edges in the fully connected GCN. Thus, EdgeScaping lays out the holistic landscape of the distribution of edges while maintaining expression intensity information derived from the GEM. This framework enables researchers to identify novel genetic relationships that cannot be strictly defined by correlation or are non-linear in nature. In the following sections we describe in detail the EdgeScaping algorithm and workflow, present results from EdgeScaping applied on five tumor sub-types dataset, and discuss the utility and limitations of the approach.

## Materials and methods

**Fig 1:**
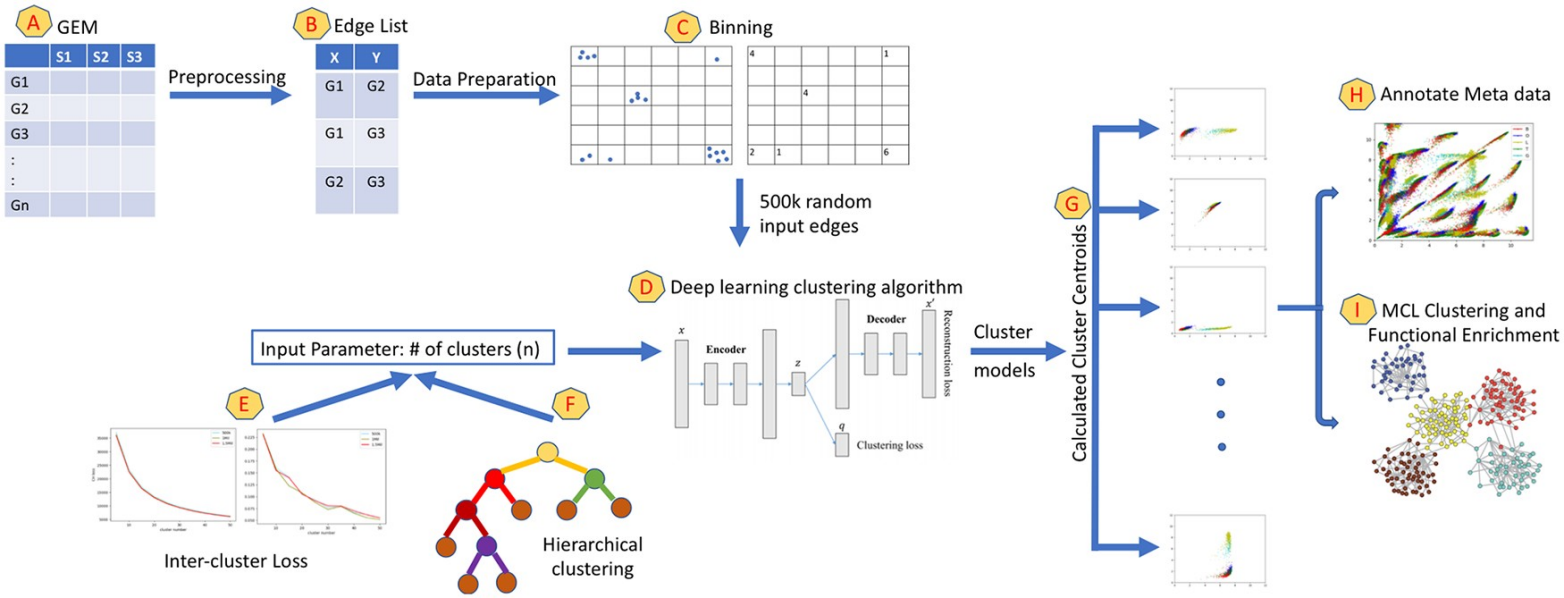
EdgeScaping workflow. Key stages of the algorithm are shown in steps A-I.

In this section we outline the EdgeScaping workflow depicted in Fig 1. Source code is available under the MIT liscense at https://github.com/bhusain/EdgeScaping under the MIT license.

### Gene Expression Matrix (GEM)

The input dataset for our algorithm was a GEM constructed from combining five cancer sub-types represented by module A in Fig 1. To construct the GEM, all normalized isoform datasets for lower grade glioma (LGG) with 534 samples, thyroid cancer (THCA) with 572 samples, glioblastoma (GBM) with 174 samples, ovarian cancer (OV) with 309 samples, and bladder cancer (BLCA) with 427 samples were obtained from The Cancer Genome Atlas [17]. The GEM contained 73,599 transcript quantifications (UCSC kg5 identifiers) making the dimensions resulting in a 2,016 row X 73,599 column GEM. The RNA sequence (RNAseq) obtained was in the Reads Per Kilobase of transcript, per Million mapped reads (RPKM) format, which required further normalizing before it was ready for analysis. Normalizing the GEM involved four steps: (a) replacing missing values with ‘NA’, (b) log2 transformation of the expression values, (c) KS-test for outlier removal (no outliers were found), and (d) quantile normalization to ensure suitable comparison between samples.

From the normalized GEM, we further performed preprocessing as depicted by module B in Fig 1 to eliminate transcripts that fall below the required expression level threshold, that is their expression levels across samples were too low to account for any significant contribution towards the network. Therefore, we dropped all transcripts that had 1500 or more (75%) samples with expression levels below 0. The number of transcripts was then reduced to 59,111 transcripts with a total of 1,747,025,605 pairwise gene comparisons (edges) across 2,016 tumors.

### Feature compression (binning)

Since there are over 1.7 billion possible edges, the entire dataset becomes computationally heavy. In GEMs with ever growing sample size and billions of edges even simple correlation calculations is cumbersome and require significant time and hardware resources. This limitation also hinders exploration of non-linear relationships within gene-gene edges. Therefore, in order to overcome the increasing dimensionality problem that is pervasive in GCN construction as datasets only grow in size, we implemented a novel solution of data compression (Binning) while still maintaining the feature information per edge. Binning converts a 2 dimensional array containing gene expression data for a gene-gene edge into a grayscale image, as depicted by module C in Fig 1. The range of the data was selected as min = 0 (since only positive expression levels are considered) and max = 19 (since the maximum expression level in the entire dataset was 18.98). Hence, each edge data was binned into 19 X 19 equal sized gene expression intensity bins. The value of each bin was calculated by the number of samples assigned to that bin based on their expression levels, therefore the range of values would always be between 0 to 2,016 and the number of bins are 361 (19*19). This reduced the number of data points that are considered per edge from 2016 x 2 to 361. Hence, each grayscale image of size 19 X 19 becomes a data point that represents a gene-gene edge.

### Deep learning clustering algorithm

In order to cluster over 1.7 billion edges we required a robust model that was able to surmise deep feature representation that included non-linear patterns. Deep clustering is one such example of building models based on learning embedded features and perform clustering by defining a clustering loss. We chose the Improved Deep Embedded Clustering (IDEC) algorithm [21] to account for data structure preservation. IDEC algorithm operates in two steps, where the first step applies an autoencoder to learn embedded features and performs dimensionality reduction, and optimizes over reconstruction loss. The second step performs clustering using the learned features and optimizes over clustering loss. The two steps are performed iteratively until the algorithm converges. Using the deep clustering algorithm of IDEC as depicted by module D in Fig 1, for a user specified parameter *n = Number of clusters*, cluster models were generated with 0.5 million, 1 million, and 1.5 million randomly selected data points from the edge list.

### Model selection

An integral component of mapping the spatial distribution of gene edge expression levels involves determining the number of clusters that are optimal for classification. To this end, we ran two tests to determine the number of clusters *n* required to train the EdgeScaping model.

**Fig 2:**
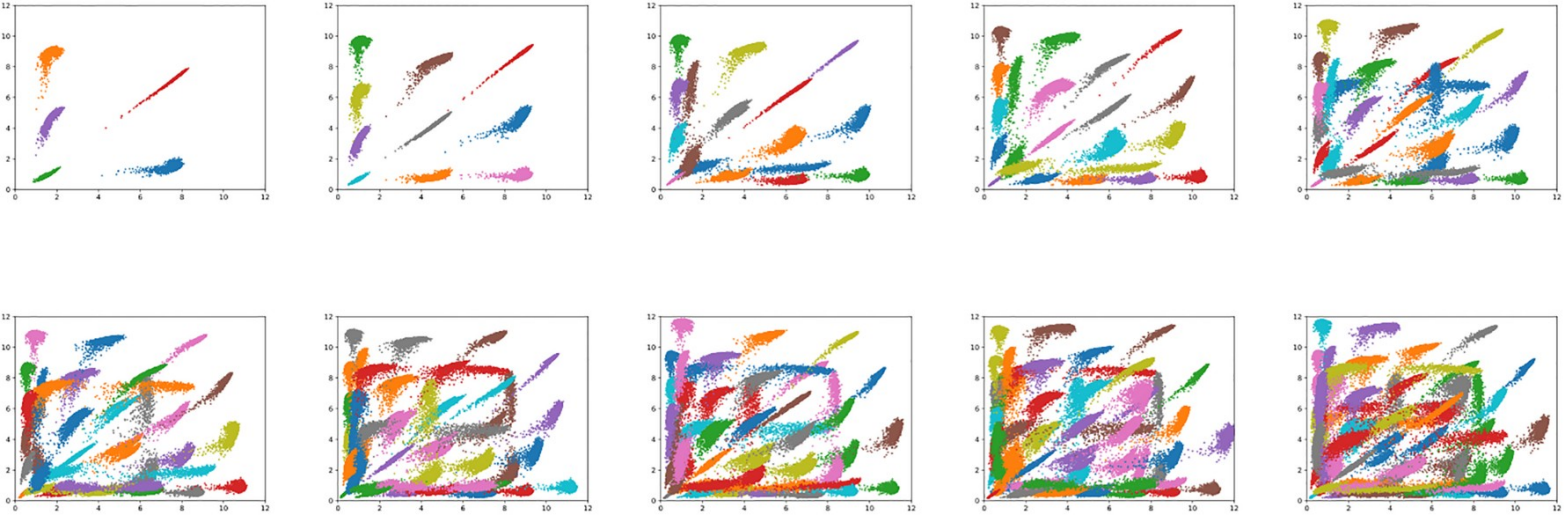
Edges sorted across a range of cluster sizes. Cluster centroids are depicted on each figure with *n* ranging between 5 to 50 with a step of 5. Horizontal and vertical axes represent log2-transformed RNA expression intensity. Each cluster was randomly assigned a color.

**Fig 3:**
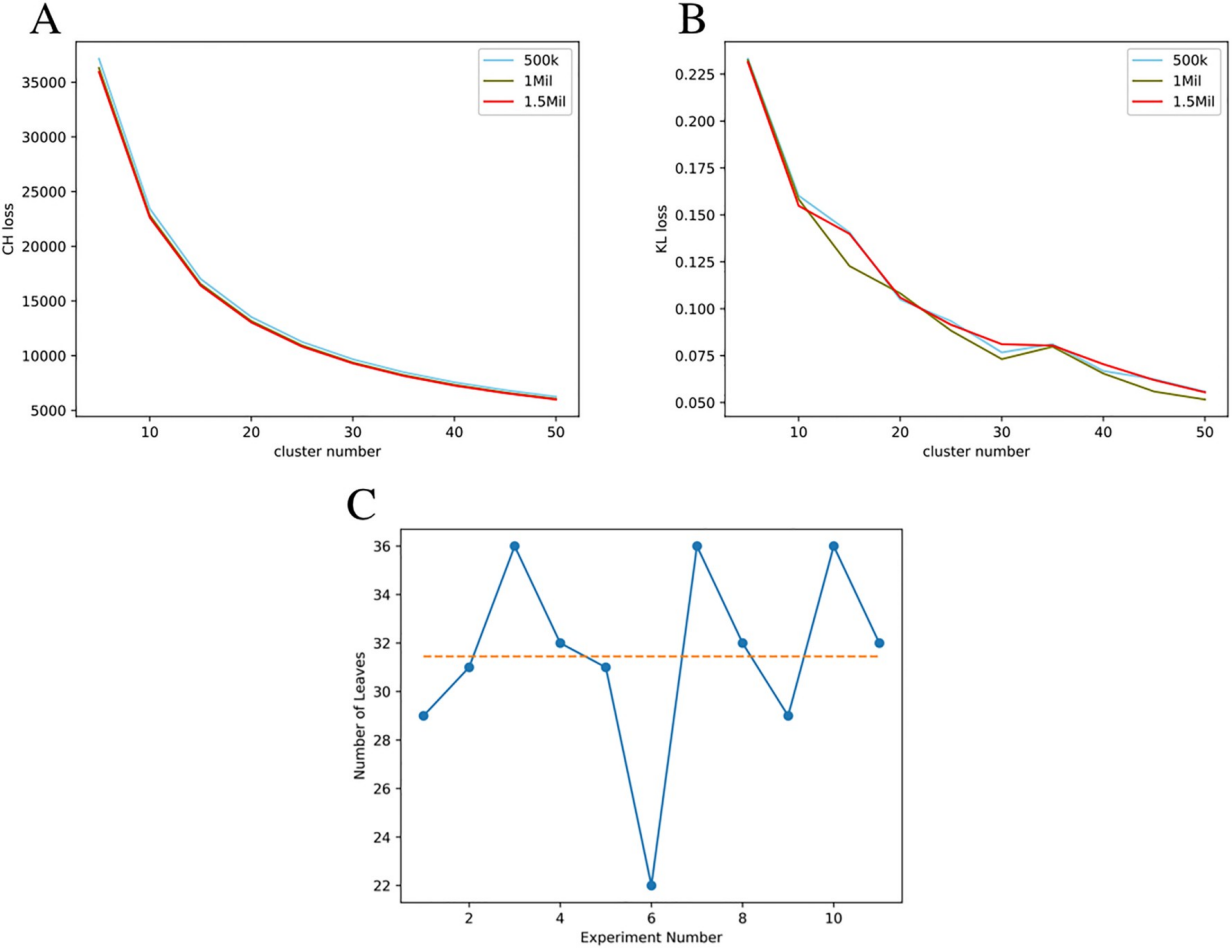
Estimating the number of co-expression clusters. Three methods were used to estimate the number of EdgeScaped clusters. (A) Calinski and Harabaz inter-cluster CH loss. CH loss value mapped between clusters for number of clusters ranging from 5 to 50 with the increment of 5. Loss value calculated for 500 thousand, 1 million, and 1.5 million sample data points. (B) Inter-cluster Silhouette Coefficient. Silhouette Coefficient mean value mapped between clusters for number of clusters ranging from 5 to 50 with the increment of 5. Loss value calculated for 500 thousand, 1 million, and 1.5 million sample data points. (C) Hierarchical clustering. Number of leaves obtained with 11 experimental iterations.

- Inter-cluster loss: The first test depicted by module E in Fig 1 to estimate *n* (number of clusters) was to calculate inter-cluster loss between centroids of the cluster models using two techniques detailed below for a wide range of *n*. We select *n* ranging from 5 to 50 with a step of 5 as depicted by Fig 2. We next plotted the calculated inter-cluster loss for each *n* to determine the plateau point for the loss values as depicted in Fig 3.
  The two loss functions used to calculate inter-cluster loss were the Calinski and Harabaz (CH) [22] score and Mean Silhouette Coefficient [23] represented by Fig 3A and 3B, respectively. The progressing loss values were collected for clusters between *n = 5* and *n = 50* with a step size of 5. Observing Fig 3, it can be noted that a plateau is reasonably achieved between cluster numbers *n = 30* and *n = 40*.
- Hierarchical clustering: The inter-cluster loss plots show the trend of loss numbers that give an overview of number of clusters that may be desirable to describe the space of expression levels. In order to select an optimum specific *n*, we ran IDEC deep clustering as an hierarchical clustering algorithm. In this experiment we began with the 500k data points and performed binary clustering hierarchically, where every branch was further split into two branches only if the inter-cluster loss (Mean Silhouette Coefficient) of that branch was greater than 10% than its parent branch. We continued the algorithm until no further splits were possible and the number of leaf nodes represented the number of clusters. Averaging the number of leaves over 11 test runs depicted in Fig 3C gave us with 32 clusters. Based on these two tests, along with visual observations of the clustering models between 5-50 clusters, we determined *n = 32* clusters to be a reasonable number to represent the classification of edge space shown in module F in our workflow depicted in Fig 1.

In Fig 3 it can also be observed that we ran the inter-cluster loss experiment for the datasets containing 500k, 1 million, and 1.5 million edges. In all three cases we observed a very similar trend. This indicates that increasing the number of data points in the training of the model did not lead to a change in the classification output, and therefore we assume that the model using 500k edges was sufficient representation of the edge space.

### Edge classification

**Fig 4:**
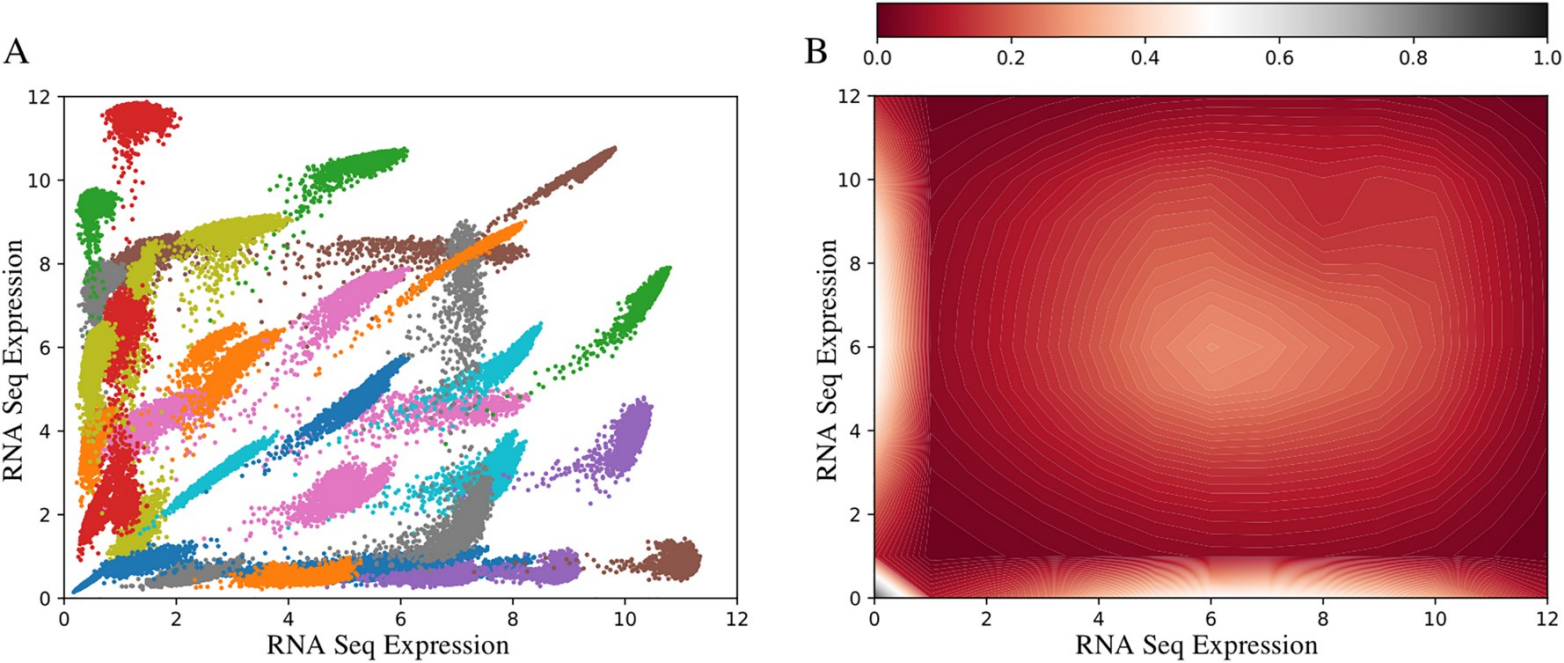
EdgeScape map for five 2,016 human tumor samples. (A) The representative centroids for n = 32 clusters depicted on the same plot. (B) Variation plot for the distribution of sample over the space of all possible gene expression levels.

**Fig 5:**
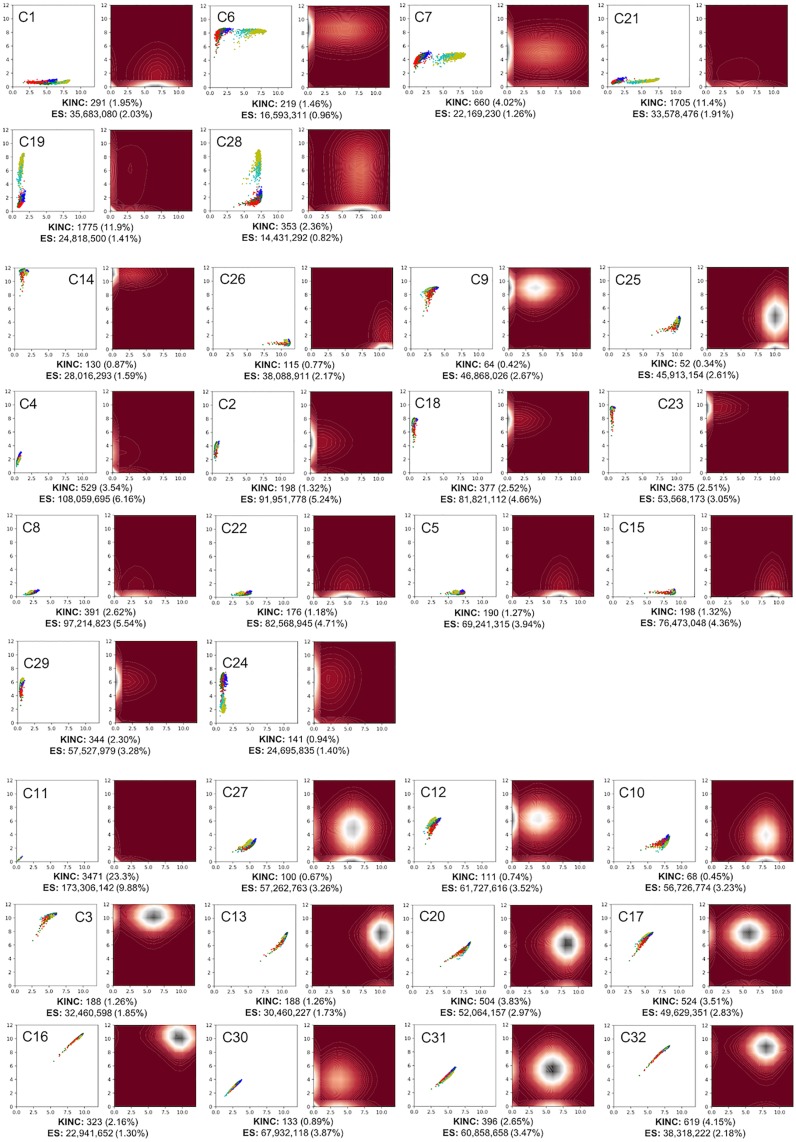
EdgeScape clusters for 2,016 human tumor samples. Each subfigure on the left depicts one of the representative cluster centroids with the cluster number (C1-C32) on the top left corner where colors indicate the five tumor sub-types. Subfigures on the right represents the variation plot for all the edges that are classified to the cluster on the left. The number of KINC and EdgeScaping (ES) edges and percent of total possible edges are shown below each cluster. Horizontal and vertical axes represent log2-transformed RNA expression intensity.

By selecting the number of clusters to classify the entire dataset as *n = 32* with the models generated using 500k samples, we classified all the 1,747,025,605 edges each falling into one of the 32 clusters. The final representative centroids of each of the 32 clusters was then calculated by averaging the value for each of the 2,016 samples for edges that were classified into that cluster. Fig 4 depicts the calculated centroids of all the clusters displayed relative to each other. Fig 5 represents each cluster centroids individually. These figures essentially depict the distribution of the pairwise (edge) gene expression intensity space for the input GEM, i.e EdgeScaping.

## Results

All potential edges were sorted using EdgeScaping into their respective 32 clusters (C1-C32) based upon pairwise RNA expression intensity. The details of the algorithm explaining why this number of clusters was selected and how the edges were sorted are discussed in the Materials and Methods. In this section, we describe the analysis of the 32 clusters of 5 tumor edges using a combination of visualization and analytic techniques to ascertain if it is possible to identify interesting edges.

### EdgeScaping human tumors

Fig 5 depicts the number of edges that are classified into expression intensity bins for each of the 32 clusters using our previous work for GCN construction (KINC algorithm) [11] and our EdgeScaping algorithm, along with their percentage representations per cluster. There are several observations apparent with the distributions of the number of edges. Notably cluster C11 represents a significant number of edges in both the KINC (23.3%) and EdgeScaping (9.88%) algorithms. This cluster specifically encapsulates edges where both genes exhibit low expression levels.

**Fig 6:**
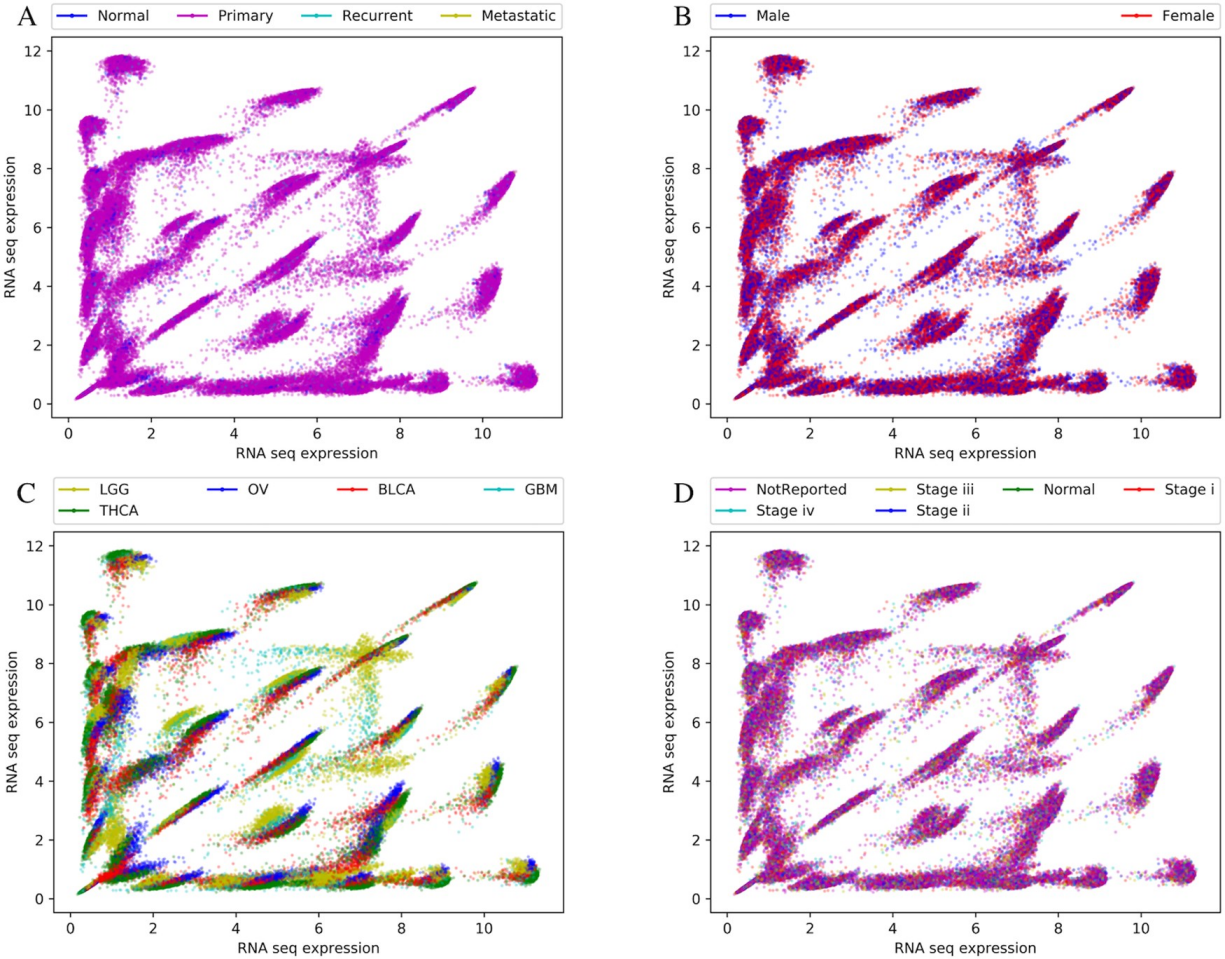
Clinical metadata mapped to EdgeScaped clusters. Representative cluster centroids are shown annotated with four classes of clinical metadata. (A) Tissue Type: Primary = primary tumor, Normal = normal tissue, Recurrent = recurrent tumor, Metastatic; (B) Gender: male, female; (C) Tumor sub-type: BLCA = bladder, OV = ovary, LGG = low-grade glioma,THCA = thyroid, GBM = glioma; (D) Tissue stages: i-iv, solid tissue Normal; NT (Normal tissue). Horizontal and vertical axes represent log2-transformed RNA expression intensity.

Fig 6 depicts metadata annotated over the distributions of the expression levels for the 32 clusters. Fig 6(A) represents each sample labeled based on different types of tissues (Primary tumor(PT), Recurrent tumor(RT), Normal tissue(NT), or Metastatic(M)). It can be observed that there were no distinct differences in the distribution based on tissue types. Similarly, it can be observed in Fig 6(B) and 6(D) that represent distribution annotated based on gender and different stages of cancer (stage i-iv, normal tissue, and not reported data) respectively, also do not show any observable distinction of interest in their expression levels. The Fig 6(C) visualizes the data with the cancer sub-type annotation involving the 5 cancer types (LGG, THCA, OV, BLCA, GBM). For multiple clusters (C1, C6, C7, C19, C21, C28), as observed in Fig 5, the scatterplot clearly depicts different pairwise expression levels for LGG and GBM (brain cancer) samples than the other cancer sub-types which is detailed in the Discussion section.

### Bimodal cluster evaluation

In order to further explore the phenomenon observed in the clusters with distinctly different expression level for brain (LGG and GBM) cancer samples in comparison to the other cancer sub-types, we isolated genes that are primarily classified into one of the 6 clusters of interest (C1, C6, C7, C19, C21, C28). This results is 5,352 genes of interest. We further explore these genes by building a GCN with all edges formed by these specific 5,352 genes that were also classified into the 6 clusters. This resulted in a GCN with 1,048,575 edges. Each edge was weighted with the distance between the nodes using the Euclidean distance measure of gene expression levels.

### MCL clustering

We utilized the Markov Cluster Algorithm (MCL) [24] to cluster the network formed using the gene expression level distances. Clustering on nodes of the GCN was performed in order to create modules in the network of genes that represent similar gene expression levels across the samples. In this particular case since we begin with genes exhibiting bimodality during EdgeScaping, we hypothesized that these genes and in extension certain modules exhibit certain behaviors more representative of brain or brain cancer functionality. The output of MCL clustering on the 1,048,575 edges resulted in 14 MCL clusters where 8 clusters formed with 5 or less genes and were not further characterized. Of the remaining 6 clusters, 3 resulted in statistically significant functional enrichment and are listed below in Table 1

**Table 1:** Functional enrichment analysis.

Gene Set	GO-MF	GO-CC	GO-BP	Pathway	Diseases
Bimodal	69	108	273	71	46
MCL Cluster 1	24	53	151	10	12
MCL Cluster 2	31	75	212	22	19
MCL Cluster 3	2	1	1	4	0
Random	0	0	0	0	0

### Functional enrichment

We performed functional enrichment analysis based on functional annotations and protein interactions network using the ToppGene suite [25]. Multiple groups of clustered gene were analyzed to observe any functional clustering concomitant to specifically examine the bimodal genes from “brain-shifted” clusters as listed in Table 1. We evaluated the number of enriched terms for five categories of gene groups: All genes involved in bimodal clusters in EdgeScaping (5,352 genes), MCL Cluster 1 (2,459 genes), MCL Cluster 2 (1,467), MCL Cluster 3 (552), and a random set of genes (1,700). Per gene set, we performed functional enrichment and counted the number of statistically significant enriched GO terms (q <10^-3^) for each of the the GO categories: Molecular Function (MF), Biological Process (BP), and Cellular Component (CC). ToppFun Pathway and Disease labels were also analyzed. Note the presence of enriched terms in the genes sets from clusters but not a random gene set. All enriched terms can be found in S1 Table.

### Overlap with known co-expression edges

In our previous work KINC utilizes the approach of Gaussian mixture models (GMMs) to construct a condition-annotated GCN for the same five tumor sub-types obtained from The Cancer Genome Atlas. That approach was specifically designed to address natural extrinsic variation during network construction from mixed input conditions. With the hypothesis that gene expression relationships exhibit modality, the GMMs allows for the identification of multiple mode for each pair-wise gene expression. The constructed GCN showed that this technique discovered tumor sub-type specific significant gene co-expression patterns (and modules) that are significantly enriched for clinical attributes.

We extended the results obtained in [11], by performing edge enrichment on the KINC constructed GCN for the five tumor sub-types and a significance threshold of p value < 0.001. Out of the total 14,908 KINC GCN edges, BLCA was enriched for 1496 edges, OV for 1986, THCA for 1353, GBM for 4414, and LGG for 8168. It is noteworthy to observe that all edges enriched for GBM and LGG were also present within the bimodal cluster GCN for EdgeScaping. We also mapped previously described KINC edges to the 32 EdgeScape clusters to observe the distribution of KINC edges over the clusters as detailed in Fig 5.

## Discussion

The formulation of GCNs allows us to explore condition-specific genetic subnetworks responsible for the biochemical mechanisms controlling phenotype expression. However, the increasing temporal and computational restrictions to determine gene-gene correlations in the presence of diluting noise sources can result in a GCN that is not fully representative of the potential gene relational network. Conventionally, these restrictions also lead to ignoring non-linear relationships that exists between genes. It is highly likely that biologically relevant edges may be ignored in the formulation of a GCN and certain irrelevant edges maybe included. In either of those cases, it is not possible to account for what the fully interconnected expression pattern of the entire gene space represents.

Our EdgeScaping algorithm addresses these limitations by quickly classifying the entire edge space of genes based on their expression intensity level using dimensionality reduction by converting sample input set into binned image data. Utilizing the algorithm, we can determine the number and types of edge clusters that exist within the dataset. As depicted in Fig 4, all 32 centroids of the classified edges are represented in the same plot. This representation of the entire space of gene edges is a holistic view of linear, non-linear, and uncoupled gene co-expression.

Our binning technique is an integral contribution towards improving the speed of sorting the edges as compared to our previous work, KINC, and is inspired by the image analysis models implemented in [26–28]. EdgeScaping uses pixelated data (images) and feeds it into the deep neural network to detect relevant features. Although there is some existing work in the field of bioinformatics that leverages image analysis along with deep neural networks to build a classification model, they nearly all primarily utilize existing images as input data [29–32], and almost none of the techniques utilize image structuring to reduce the dimensionality.

A key feature of our approach is that converting scatterplot of gene edges to binned images is highly scalable. Even when the number of samples increase from 2,016 to a magnitude multiple times its size, the only effect on the overall computation time is during the binning step of the algorithm. This addresses a critical bottleneck that GCN algorithms face with ever increasing size of GEMs. Another essential contribution of our technique by conversion of data from array of numbers into an image format is that it allows us to leverage vast advancements made in the field of deep learning and image analysis to discover patterns and hidden non-linear relationships between genes. This novel representation of gene-gene edge data as images permits for exploration of network regulation and interaction using modalities that have not been previously explored. In EdgeScaping we demonstrate one such application of edge analysis that permits us to classify a fully connected GCN in a fast and efficient manner to explore patterns that were previously left undetected.

### Is this all noise?

One major concern about EdgeScaping classification is that a significant number of edges probably do not exhibit any interesting genetic relationships yet still get classified into clusters. We have in essence characterized all the signal and noise in the dataset leading to the important question: Are EdgeScape networks mostly noise? The simple answer is yes. EdgeScaping does include the millions of noisy edges when constructing the map of pairwise gene expression. In fact, an EdgeScaped GEM is defined by what the noisy edges of the dataset look like and how they are distributed. However, it is useful to determine the entire edge space while tracking pairwise gene expression intensity before designing the restriction criteria that eliminate the uncorrelated edges. This is especially true when one wants to account for dependent edges that were not found to be correlated via traditional correlation metrics (e.g. Spearman, Pearson) that ignore non-linear polynomial relationships.

Even in the face of noisy edges, there is evidence that our approach sorts edges into clusters with variable correlation signal as observed in Fig 5. For example, clusters C11, C19, and C21 collectively contain 46.6% of the previously described KINC edges suggesting that these intensity-binned edge clusters are enriched for more significant GCN edges relative to the other clusters. Further, these known KINC edges are now placed in a pairwise gene expression intensity context. Since specific EdgeScaped clusters are enriched for significant correlation, these clusters can be prioritized for GCN construction thereby significantly reducing the computational time required to sort through noisier clusters. Hence EdgeScaping can be utilized as a quick and efficient preprocessing technique.

EdgeScaping clusters also identified signal through the noise at the condition level. For example, consider cluster C6 in Fig 5 which is annotated with metadata from different tumor sub-types. It is apparent that the samples associated with LGG and GBM tumors contain a distinct shift in co-expression level when compared to other tumor sub-types (OV, BLCA, and THCA) thus making cluster C6 a bimodal cluster. Similar bimodal clusters can also be observed in clusters C1, C7, C21, C19, and C28 with a distinct expression intensity shift for LGG and GBM indicating that there is a strong relationship between edges that were classified into these clusters and pathways that control brain and/or brain tumor function.

### Evaluating bimodal clusters

In order to test if the edges classified within the bimodal clusters depicting the clear shift in LGG and GBM expression patterns may exhibit brain and/or brain tumor function, we examined the collective function of 5,352 genes in bimodal clusters (C1, C6, C7, C19, C21, C28) that involved a total of 1,048,575 edges. By applying MCL clustering and analyzing the significant edges, it can be observed from Table 1 that a statistically significant number of bimodal genes were enriched for GO, pathway, and disease terms, including the MCL cluster1 and MCL cluster2 subsets. In contrast, we experimented with 1,700 random genes with multiple test runs and did not find these random genes to be statistically significant for any function. The results of term enrichment for Table 1 can be found in S1 Table.

On closer inspection of enriched terms for MCL cluster1, it was found that bimodal cluster edges, while containing samples for five tumor sub-types, were enriched for brain function attributes as opposed to hallmark cancer processes as depicted in Table 2. The GO biological process, GO cellular component, and Pathway terms included ‘synapse’, ‘neuron part’, ‘trans-synaptic signaling’, ‘chemical synaptic signaling’, ‘neurogenesis’, ‘neuron projection’, ‘neuronal system’, and other related terms. Furthermore, by observing the category enriched for diseases it is clear that significant terms lean more towards brain related diseases but not brain cancer. Our future work involves further investigating the set of genes extracted via bimodal clustering that are more consistent with tumor versus brain biology. Overall, based on Tables 1 and 2 it seems very likely that for this GEM, the bimodal clusters are non-randomly associated with specific tumor sub-types thus enabling a novel path to condition-specific edge-oriented biomarker discovery.

**Table 2:** Top five enriched functions for genes in cluster 1.

Category	Term ID	Term Description	q-value
GO-MF	GO:0008092	cytoskeletal protein binding	1.52E-07
	GO:0005085	guanyl-nucleotide exchange factor activity	1.28E-05
	GO:0030695	GTPase regulator activity	1.28E-05
	GO:0005088	Ras guanyl-nucleotide exchange factor activity	4.74E-05
	GO:0060589	nucleoside-triphosphatase regulator activity	7.98E-05
GO-CC	GO:0045202	synapse	3.82E-34
	GO:0097458	neuron part	1.99E-33
	GO:0043005	neuron projection	8.23E-28
	GO:0044456	synapse part	8.31E-28
	GO:0044463	cell projection part	4.65E-21
GO-BP	GO:0098916	anterograde trans-synaptic signaling	1.94E-20
	GO:0007268	chemical synaptic transmission	1.94E-20
	GO:0099537	trans-synaptic signaling	1.94E-20
	GO:0099536	synaptic signaling	3.04E-20
	GO:0032990	cell part morphogenesis	3.70E-18
Pathway	1268763	Neuronal System	2.01E-09
	169346	Regulation of RAC1 activity	1.29E-06
	1268766	Transmission across Chemical Synapses	9.25E-06
	P00057	Wnt signaling pathway	1.34E-03
	1270303	Axon guidance	1.34E-03
Diseases	C0036341	Schizophrenia	2.27E-09
	C0005586	Bipolar Disorder	1.32E-07
	C3714756	Intellectual Disability	1.32E-07
	C0004352	Autistic Disorder	7.52E-06
	C0014544	Epilepsy	1.12E-05

Another interesting observation is the significant number of KINC edges observed within the bimodal clusters, especially cluster C19 (11.9%) and C21 (11.4%). This indicates that a substantial number of KINC edges are found within the bimodal clusters as opposed to clusters with different distributions. This phenomena can also be observed by performing edge enrichment on KINC edges where it was observed that out of the total 14,908 edges, 4,414 edges were enriched for GBM and 8,168 for LGG, further indicating a significant amount edges that were brain or brain tumor related.

### EdgeScaping efficiency

A core issue of clustering more than 1.7 billion edges within realistic computational and time constraints was the requirement that the algorithm be able to efficiently and quickly create the model as well as cluster the edges. We addressed this requirement by leveraging the advancements in speed and accuracy demonstrated by deep neural network clustering. In order to adapt our data into input to the IDEC clustering algorithm, we transformed the gene-pair samples into a reduced dimensionality image based dataset. This allowed us to work with binned data that is significantly smaller and therefore processes faster than the original dataset. Further, the EdgeScaping framework facilitates the exploration of new deep learning tools and techniques that are constantly being refined.

### Limitations

Although, the conversion of edge data into an image format will always perform dimensionality reduction and make analysis scalable for increasing GEM sizes, we note there may be conditions where the noise of the entire GCN overshadows the underlying signals. In this paper we have demonstrated a case study as to why exploring the entire landscape of the GCN might lead to detection of hidden polynomial relationships, there may be cases where categorization of all the edges may not lead to easily identifiable patterns.

### Future work

There are two directions in which this research will move forward. Firstly, due to the efficient speed in clustering over 1.7 billion edges, we can now transform huge GEMs with increased number of samples which can be easily converted to binned expression data bound by the minimum and the maximum expression values of the GEM. This ensures that the computation time for model generation and clustering will not increase with the increase in the number of samples. Hence, future works will include application of EdgeScaping algorithm on panCancer TCGA matrix that comprises of 33 cancer sub-types which can be annotated and analyzed for various types of metadata. The second set of experiments are aimed towards further exploring the candidate biomarker edges that were identified via EdgeScaping to be associated with brain tumors yet appear to be enriched for non-tumor function.

## Conclusion

In this paper, we detailed a technique that reveals the holistic edge space derived from the GEM, modelled as an image, rather than a select subset of significant edges we “landscape” the entire range of pairwise gene expression relationships. EdgeScaping allows one to map gene pairs in a quick, efficient manner using a dimensionality reduction binning technique and deep learning algorithm. Annotating metadata over the clustered centroids allows one to visualize patterns that were not apparent using other conventional techniques that create GCNs. Through this approach, distinct patterns were identified for brain tumor sub-types to identify potential biomarker genes and edges for these biological conditions.

## Supporting information

S1 Table Full functional enrichment analysis of bimodal cluster genes. (TXT)
